# Biochanin A prevents neurodegeneration and oxidative stress in a kainic acid model of epilepsy by activating the PI3K/Akt/Nrf2 signaling pathway

**DOI:** 10.1038/s41598-025-23414-z

**Published:** 2025-11-13

**Authors:** Ratchaniporn Kongsui, Tichanon Promsrisuk, Teera Chanmanee, Lars Klimaschewski, Sataporn Jamsuwan, Napatr Sriraksa, Jinatta Jittiwat, Sitthisak Thongrong

**Affiliations:** 1 Division of Physiology, School of Medical Sciences, University of Phayao, Phayao, 56000 Thailand; 2 Division of Anatomy, School of Medical Sciences, University of Phayao, 19 Moo 2, Tambon Maeka, Amphur Muang Phayao, Phayao, 56000 Thailand; 3 Division of Neuroanatomy, Department of Anatomy Histology and Embryology, Innsbruck Medical University, 6020 Innsbruck, Austria; 4 Faculty of Medicine, Mahasarakham University, Maha Sarakham, Maha Sarakham 44000 Thailand

**Keywords:** Biochanin a, PI3K/Akt/Nrf2 signaling pathway, Antioxidants, Kainic acid, Epilepsy, Neuroscience, Anatomy, Molecular medicine

## Abstract

Epilepsy is a serious neurodegenerative disorder, which affects approximately 50 million people worldwide. Growing evidence indicates that oxidative stress caused by an excessive production of free radicals is considered one of the etiologies of epilepsy. Several studies have demonstrated that neurotoxicity-induced oxidative stress leads to neuronal cell death and seizure severity. In this study, we investigated the possible effects of biochanin A (BA), a natural isoflavone containing antioxidant and neuroprotective activities, in a mouse model of epilepsy, induced by an intraperitoneal injection of kainic acid (KA). The administration of KA produced severe seizure activity, and a decrease in memory function. Histological studies following KA injection revealed significant neurodegeneration, astrocyte dysfunction, and microglia activation in CA1, CA3, and hilus of the hippocampus. Moreover, KA administration exhibited an increase in caspase-3, a decrease in antioxidative enzyme levels, and downregulation of the PI3K/Akt/Nrf2 signaling pathway. Conversely, BA treatment significantly increased seizure threshold and memory function. In addition, BA treatment reduced neuronal death, restored astrocyte function, and suppressed microglia activation in CA1, CA3, and hilus. The BA-mediated neuroprotection was accompanied by the significant upregulation of a crucial cellular pathway particularly related to the antioxidant defense system and cell survival, the PI3K/Akt/Nrf2 signaling pathway, thereby increasing antioxidant enzyme levels (GSH, SOD, CAT) and reducing neuronal death. Taken together, our results suggest that BA possesses a neuroprotective effect through bolstering antioxidant defense mechanisms and activating neuronal survival. Thus, BA could be a possible pharmacological agent for the amelioration of epilepsy or other neurodegenerative diseases where oxidative stress is a key factor.

**Supplementary Information:**

The online version contains supplementary material available at 10.1038/s41598-025-23414-z.

## Introduction

Excitotoxicity plays a major role in neuronal degeneration associated with various neurodegenerative diseases such as Alzheimer’s disease (AD), Parkinson’s disease (PD), amyotrophic lateral sclerosis (ALS), and epilepsy^1^. Kainic acid (KA), an agonist of AMPA/kainate receptor subtypes of ionotropic glutamate receptors (iGluRs), is widely used in numerous studies to induce status epilepticus and hippocampal tissue damage in animal models of temporal lobe epilepsy^2–4^. It is well known that administration of KA triggers an overstimulation of iGluRs resulting in neuronal membrane depolarization, which causes excessive calcium ion (Ca^2+^) influx into neurons^5^. Ca^2+^ overloading leads to mitochondrial dysfunction, which triggers the release of apoptotic enzymes including caspases and proteases, that are responsible for neuronal apoptosis^6^. Mitochondrial damage by Ca^2+^ influx also causes a massive generation of reactive oxygen species (ROS), and consequently oxidative stress-induced neuronal death^7^. Therefore, reducing apoptotic enzymes and oxidative stress following mitochondrial dysfunction caused by excitotoxicity could be a potential therapy for epilepsy.

**Fig. 1:**
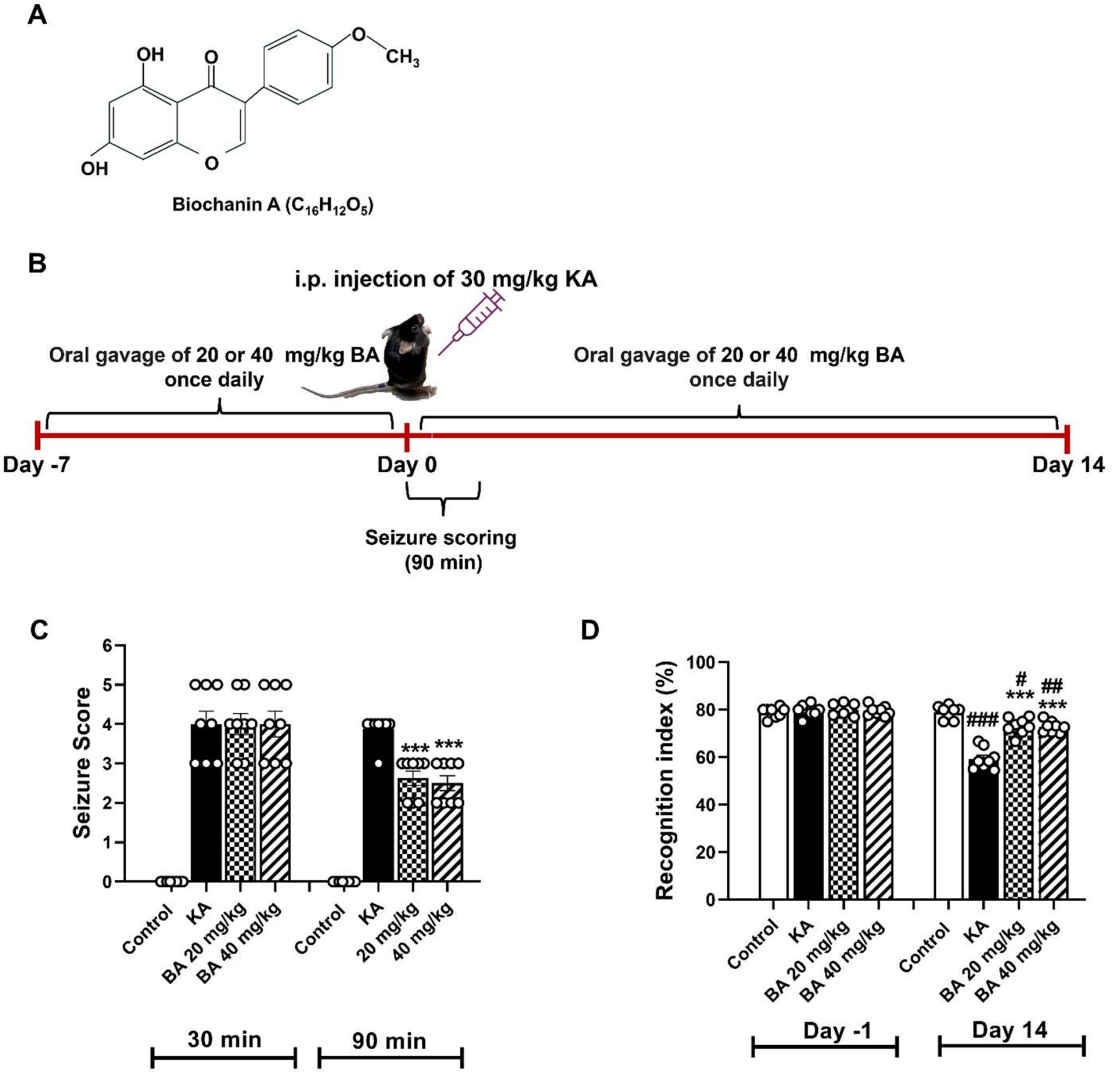
BA attenuates seizure activity and memory deficits induced by KA injection. Chemical structure of biochanin A (**A**). Experimental procedure of BA treatment and i.p. injection of KA (**B**). Seizure score was observed during a 90 min period after KA administration (**C**). Treatment with BA improved cognitive function, as indicated by an increase in the recognition index (**D**). Data are expressed as mean ± SEM (*n* = 8 mice per group). #*p* < 0.05, ##*p* < 0.01, ###*p* < 0.001 compared with control group; ***p < 0.001 compared with KA group.

Biochanin A (BA) is a natural isoflavone (Fig. 1A) mainly found in plants of the family Leguminosae or Fabaceae. BA possesses many pharmacological activities including anti-cancer activity^8^, anti-inflammatory activity^9^, antioxidant activity^10^, and neuroprotective activity^11^. Currently, numerous studies have focused on the effects of BA in neurological diseases. An in vitro study in PC12 cells showed that BA could reduce β-amyloid-induced caspase activity and mitochondrial membrane collapse in a model of AD. Moreover, BA attenuated glutamate-induced excitotoxicity by increasing the expression of glutathione levels in a cell line^12^. In a rat model of PD, BA exhibited neuroprotective effects against lipopolysaccharide-induced dopaminergic neuronal loss in the pars compacta of substantia nigra. BA inhibited microglial activation and protected dopaminergic neurons by increasing antioxidant levels including superoxide dismutase (SOD) and glutathione peroxidase (GPx), meanwhile reducing the content of malondialdehyde (MDA), a maker of lipid peroxidation^13^. Thus far, the neuroprotective mechanisms of BA against KA-induced excitotoxicity are not fully understood. Therefore, this study was aimed to explore whether BA could prevent hippocampal neuronal apoptosis and increase seizure threshold throughout the improvement of antioxidant levels in KA-induced oxidative stress in a mouse model of epilepsy.

## Materials and methods

### Kainic acid-induced seizure and biochanin A treatment

Thirty-two male C57BL/6NJcl mice (8-week-old, weighing 24 ± 2 g) were obtained from Nomura Siam International Co., Ltd, Thailand. All animal procedures were conducted with the approvement of the Animal Ethics Committee of the University of Phayao, Thailand (approval no. 1-008-67). Prior to the experiment, all mice were acclimatized for one week and maintained under standard laboratory environments with ad libitum to water and food. The experimental plan is depicted in Fig. 1B, in brief, all mice were randomly divided into four experimental groups of eight mice per group:^1^ intraperitoneal (i.p.) injection of sterile normal saline (control)^2^, KA alone^3^, KA + 20 mg/kg BA, and^4^ KA + 40 mg/kg BA.

To induce status epilepticus, KA (cat# FK46986, Biosynth Ltd, UK) was dissolved in sterile normal saline and administered via a single i.p. injection at a dose of 30 mg/kg, based on our previous study^14^. BA (purity: > 98.0%, cat# 491-80-5, Tokyo Chemical Industry Co., Ltd., Japan) was dissolved in sterile normal saline and applied via oral gavage once daily. The BA doses used in this study were chosen based on a literature review^11^. The mice in the KA + BA groups received oral gavage administration of BA at a dose of 20 mg/kg or 40 mg/kg once daily for a total of 7 days before, and were continually treated with BA for 14 days after KA administration. The control and KA alone mice received sterile normal saline as a vehicle.

### Seizure scoring

All mice were observed for seizure activities at two different time points for 90 min after i.p. injection of KA. The seizure scores were graded with the following score criteria: (0) normal behavior; (1) facial muscle clonus; (2) nodding head; (3) forelimbs clonus; (4) rearing with forelimbs clonus; (5) generalized tonic–clonic seizure including death^15^. Only mice with a score of 3–5 were included in this study.

### Novel objective recognition test (NORT)

Prolonged seizures have been reported to induce neuronal cell death in the cornu ammonis 1 (CA1) and cornu ammonis 3 (CA3) sub-fields of the hippocampus leading to memory deficit^3,4^. To investigate whether BA could improve memory function after KA administration, all mice were assigned to the novel objective recognition test (NORT) on day one before (day − 1) and day fourteen after KA injection. NORT was performed as described in the previous study^16^. On the first day, each mouse was placed in the middle of the empty open field arena made of wood-plastic composite (dimension 40 × 40 × 40 cm), and allowed to explore the arena for 5 min. On the testing day, during the training phase, two identical objects were placed in opposite quadrants of the arena. The mouse was placed in the middle of the open field arena equidistant from both identical objects, and allowed freely to explore these two objects for 5 min. Four hours after the training phase, one object was replaced by the novel object at the same location as during the training phase. The mouse was put in the center of the open field arena, and allowed free exploration for 5 min. The exploration time was recorded when nose-object contact was established. The arena and objects were thoroughly cleaned between mice using 70% ethanol to avoid interference with subsequent test results. The NORT data are presented as percentage of recognition index (RI), which was calculated using the following formula RI = [time spent exploring the novel object/(time spent exploring the novel object + time spent exploring the identical object)] × 100^16^.

### Cresyl Violet staining and immunohistochemistry

To study the neuroprotective effect of BA on KA-induced hippocampal cell death, cresyl violet staining was conducted after the completion of all experiments according to our previous protocol^14^. Briefly, five mice from each experimental group were sacrificed by an overdose of anesthetic (70 mg/kg thiopental sodium, i.p.) following by intracardial perfusion with sterile normal saline. The brains were removed and fixed in 4% paraformaldehyde in 0.1 M phosphate buffered saline (PBS) pH 7.4 for 48 h, then cryoprotected in 30% sucrose in PBS for 48 h. After that, the brains were serially cut into 20 μm coronal sections by a cryostat (Leica Microsystems, Inc., CM1950). Brain sections were mounted on positively charged microscope slides, then subjected to Nissl staining using cresyl violet dye (cat# 1.05235, Sigma-Aldrich, USA).

Immunohistochemical staining was done in free-floating sections using our established protocol^14^. In brief, the sections were incubated for 90 min at 25 °C in blocking solution (10% horse serum in PBS), and then overnight at 4 °C with primary antibodies: mouse anti-glial fibrillary acidic protein (GFAP) antibody (1:500, cat# MABN92, Sigma-Aldrich, USA) or mouse anti-ionized calcium binding adaptor molecule 1 (Iba1) antibody (1:500, cat# MABN92, Sigma-Aldrich, USA). The brain sections were then incubated with donkey biotinylated anti-mouse secondary antibody (1:500, cat# 715-065-150, Jackson ImmunoResearch, USA) for 2 h at 25 °C, followed by signal amplification using extravidin peroxidase (1:1000, cat# E2886, Sigma-Aldrich, USA) for 1 h. Thereafter, brain sections were colorized using 3,3′-diaminobenzidine (DAB) (cat# D12384, Sigma-Aldrich, USA). All images were acquired at 1.8–2.0 mm posterior to the bregma according to the mouse brain atlas^17^ using the Eclipse Ni-U upright microscope (Nikon, Japan) under 400× magnification, and analyzed with the NIS Elements imaging software version 5 (Nikon, Japan). The number of GFAP-positive cells, IBA1-positive cells, and the neurons in the CA1 and CA3 sub-fields of the hippocampus were assessed using three areas of interest from each brain section, while the number of GFAP-positive cells, IBA1-positive cells, and interneurons of the hilus were counted in the entire hilar area.

### Protein preparation

The hippocampi were collected and cleaned with PBS. The tissues were homogenized in 500 µl of lysis buffer (cat# RB4475, BioBasic, Canada) supplemented with 1% protease inhibitor cocktail set I (cat# 539131, Sigma-Aldrich, USA). Homogenized samples were stored for 30 min on ice, then centrifuged at 4 °C for 15 min at 10,000*g*. Protein concentrations were determined by Qubit protein assay kit (cat# Q33211, Thermo Fisher Scientific Inc, USA) according to the manufacturer’s protocol using the Qubit 4 Fluorometer device (Invitrogen, USA).

### Determination of antioxidant activities and lipid peroxidation

Reduced glutathione (GSH) levels were measured using our described protocols^14^. In brief, reduced GSH (cat# 70-18-8, Sigma-Aldrich, USA) was dissolved in 0.1 N HCl and used as standard GSH. Subsequently, 20 µl of the standard GSH or lysates were mixed with 250 µl of 0.1 M phosphate buffer pH 7.6, and 50 µl of 5,5′-dithio-bis-(2-nitrobenzoic acid) (DTNB) (cat# 69-78-3 Sigma-Aldrich, USA), then kept in the dark for 5 min. GSH levels were analyzed at 412 nm using a microplate reader.

Superoxide dismutase (SOD) activity was determined using SOD assay kit (cat# S311, DOJINDO, Japan) according to the manufacturer’s instruction. Briefly, 20 µl of lysates were mixed with 200 µl of WST working solution, and 20 µl of enzyme working solution, then incubated at 37 ℃ for 20 min. The SOD enzyme activities were measured by the absorbance at 405 nm using a microplate reader.

The activity of catalase (CAT) was quantified according to our described protocol^14^. Shortly, 20 µl of lysates were added to 100 µl of 6 mM H_2_O_2_, followed by an incubation at 37 °C for 1 min. The reaction was stopped with 100 µl of 32.4 mM ammonium molybdate. CAT activity was measured at 405 nm by a microplate reader.

Lipid peroxidation was quantified using the malondialdehyde (MDA) assay following a previously described protocol^18^. Briefly, 150 µL of lysates were added to 10% trichloroacetic acid and incubated at 25 °C for 10 min. Thereafter, 0.6% thiobarbituric acid was added to the solutions, which were then heated in a water bath for 30 min. The supernatants were centrifuged at 2,800 g for 20 min, and the absorbance of thiobarbituric acid-reactive substances was read at 532 nm using a microplate reader.

### Western blots

The total amounts of hippocampal proteins (20 µg) from three mice of each group were separated by 10% sodium dodecyl sulfate-polyacrylamide gel electrophoresis (SDS-PAGE) and transferred onto polyvinylidene difluoride (PVDF) membranes (cat# IPVH00010, Merck millipore, USA). The membranes were blocked with 5% skim milk in Tris-buffered saline containing 1% Tween-20 at 25 °C for 1 h, then incubated with the primary antibodies; anti-cleaved-caspase 3 antibody (1:1000, cat# AF7022, Affinity Biosciences, USA), anti-caspase 3 antibody (1:1000, cat# AF6311, Affinity Biosciences, USA), anti-pan-Akt1/2/3 antibody (1:1000, cat# AF6261, Affinity Biosciences, USA), anti-phospho-Akt1/2/3 antibody (1:1000, cat# AF0016, Affinity Biosciences, USA), anti-Nrf2 antibody (1:1000, cat# AF0639, Affinity Biosciences, USA), anti-PI3K antibody (1:1000, cat# AF6242, Affinity Biosciences, USA), anti-phospho-PI3K antibody (1:1000, cat# AF3242, Affinity Biosciences, USA) and anti-β-actin antibody (1:10,000, cat# T0022, Affinity Biosciences, USA) overnight at 4 °C. Then, the blots were incubated with the appropriate HRP-conjugated secondary antibodies; goat anti-rabbit (1:5,000, cat# AP307P, Sigma-Aldrich, USA) or goat anti-mouse (1:5,000, cat# 62–6520, Invitrogen, USA) for 2 h at 25 °C. The protein bands were visualized using enhanced chemiluminescence and detected with a chemiPRO XS Western Blot Imaging System (Cleaver Scientific, UK). Protein band densities were analyzed by ImageJ software (Pierce, Rockford, IL, USA) and normalized to β-actin.

### Statistical analysis

All experimental data were analyzed using GraphPad Prism software version 9.0. The one-way analysis of variance (ANOVA) followed by Tukey’s post hoc test was used to determine significant differences among groups, with *p* < 0.05.

## Results

### BA attenuates seizure activities and memory impairment in KA-induced epilepsy

Seizure activity was investigated at two different time points for 90 min after a 30 mg/kg i.p. injection of KA. No seizure activities developed in control mice during the 90 min observation period. After KA injection, mice exhibited seizure-like behaviors, for example, myoclonus, rearing, and falling, and seizure scores dramatically increased to 3.89 ± 0.20 in KA alone, 3.77 ± 0.20 in KA + 20 mg/kg BA, and 3.82 ± 0.20 in KA + 40 mg/kg BA at 30 min. Notably, BA treatment at doses of 20 or 40 mg/kg significantly decreased seizure scores to 2.67 ± 0.14 (*p* < 0.001) and 2.53 ± 0.13 (*p* < 0.001), respectively, compared to mice treated with KA alone (3.80 ± 0.14) at 90 min after KA injection (Fig. 1C).

Learning and memory alterations after 14 days of KA treatment were evaluated using the novel objective recognition test (NORT). KA-treated mice (59.29 ± 1.59%) exhibited a significant reduction in the recognition index (RI) compared to the control group (79.40 ± 1.01, *p* < 0.05). However, treatment with 20 or 40 mg/kg BA significantly increased the RI to 73.11 ± 1.23% (*p* < 0.001) and 72.81 ± 0.85% (*p* < 0.001), respectively, when compared to mice treated with KA alone two weeks after seizure induction (Fig. 1D).

### BA mitigates oxidative stress in KA-induced epilepsy by increasing antioxidant levels

**Fig. 2:**
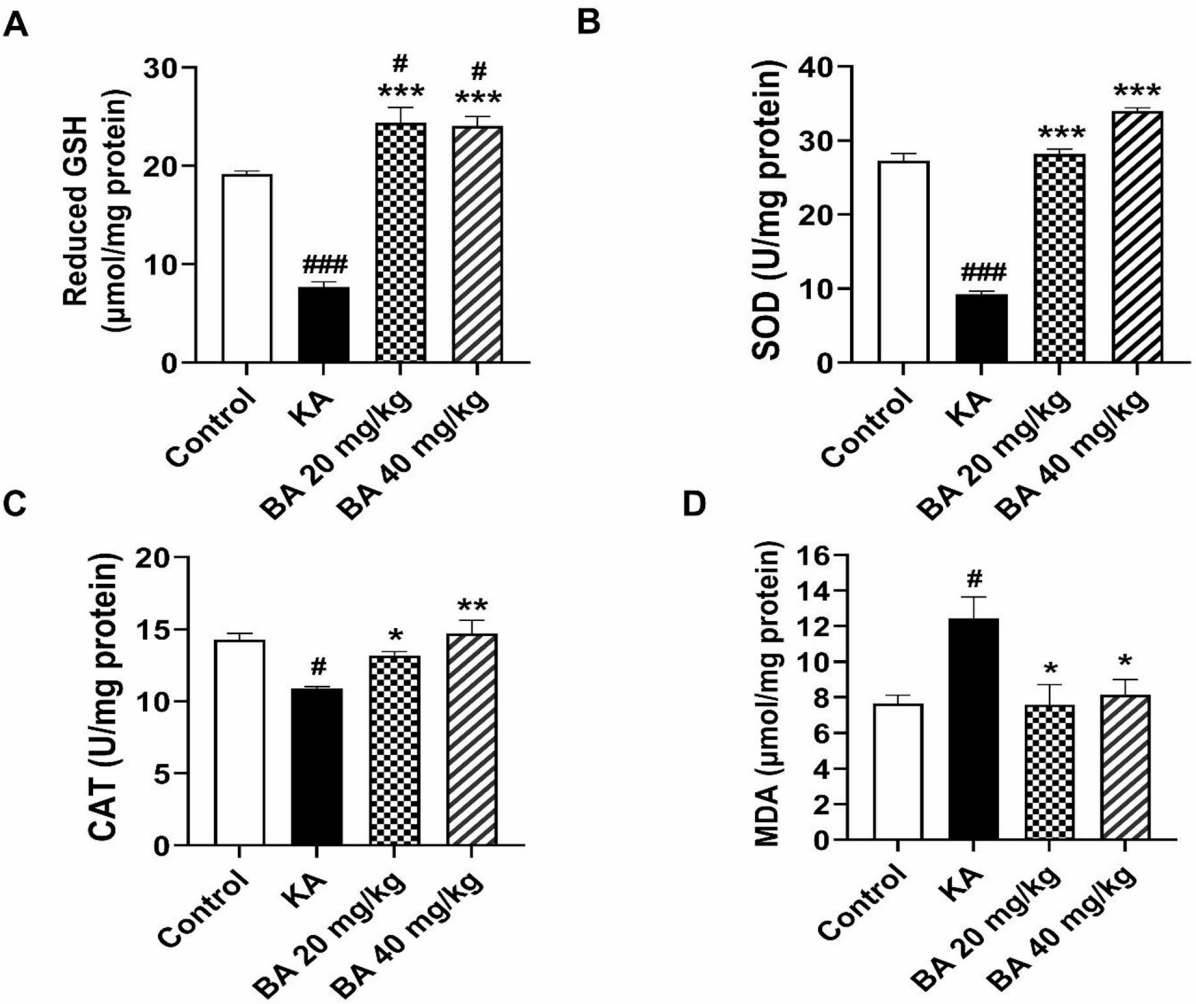
BA enhances the levels of antioxidant enzymes including GSH (**A**), SOD (**B**), and CAT (**C**), leading to a reduction in MDA level (**D**), an oxidative stress marker increased following KA treatment. Results are expressed as mean ± SEM (*n* = 4 mice per group). #*p* < 0.05, ###*p* < 0.001 compared with control group; *p < 0.05, **p < 0.01, ***p < 0.001 compared with KA group.

It is well known that treatment with KA triggers a massive production of free radicals, resulting in oxidative stress and subsequent neuronal cell death, thereby further promoting epileptic seizures^19^. Therefore, hippocampal antioxidant levels were investigated by measuring the contents of antioxidant defense enzymes including GSH, SOD, and CAT. Notably, KA administration resulted in a significant reduction in GSH, SOD, and CAT levels when compared to control and BA-treated mice. However, treatment with BA at 20 or 40 mg/kg significantly increased these antioxidant enzyme levels compared to KA alone mice (Fig. 2A, C).

In contrast to the antioxidant enzyme levels, MDA (a maker of lipid peroxidation products) was increased after KA treatment. MDA content was significantly increased in the KA alone group (12.45 ± 1.20 µmol/mg protein) compared to the control group (7.66 ± 0.47 µmol/mg protein, *p* < 0.05). Strikingly, BA treatment at 20 or 40 mg/kg significantly reversed the KA-induced MDA production compared to KA alone group (Fig. 2D). Collectively, these findings suggest that BA mitigates KA-induced oxidative stress by enhancing the antioxidant defense enzymes including GSH, SOD, and CAT, resulting in a decrease in seizure scores and neuronal cell death.

### BA protects against neuronal cell death in the hippocampus induced by KA

**Fig. 3:**
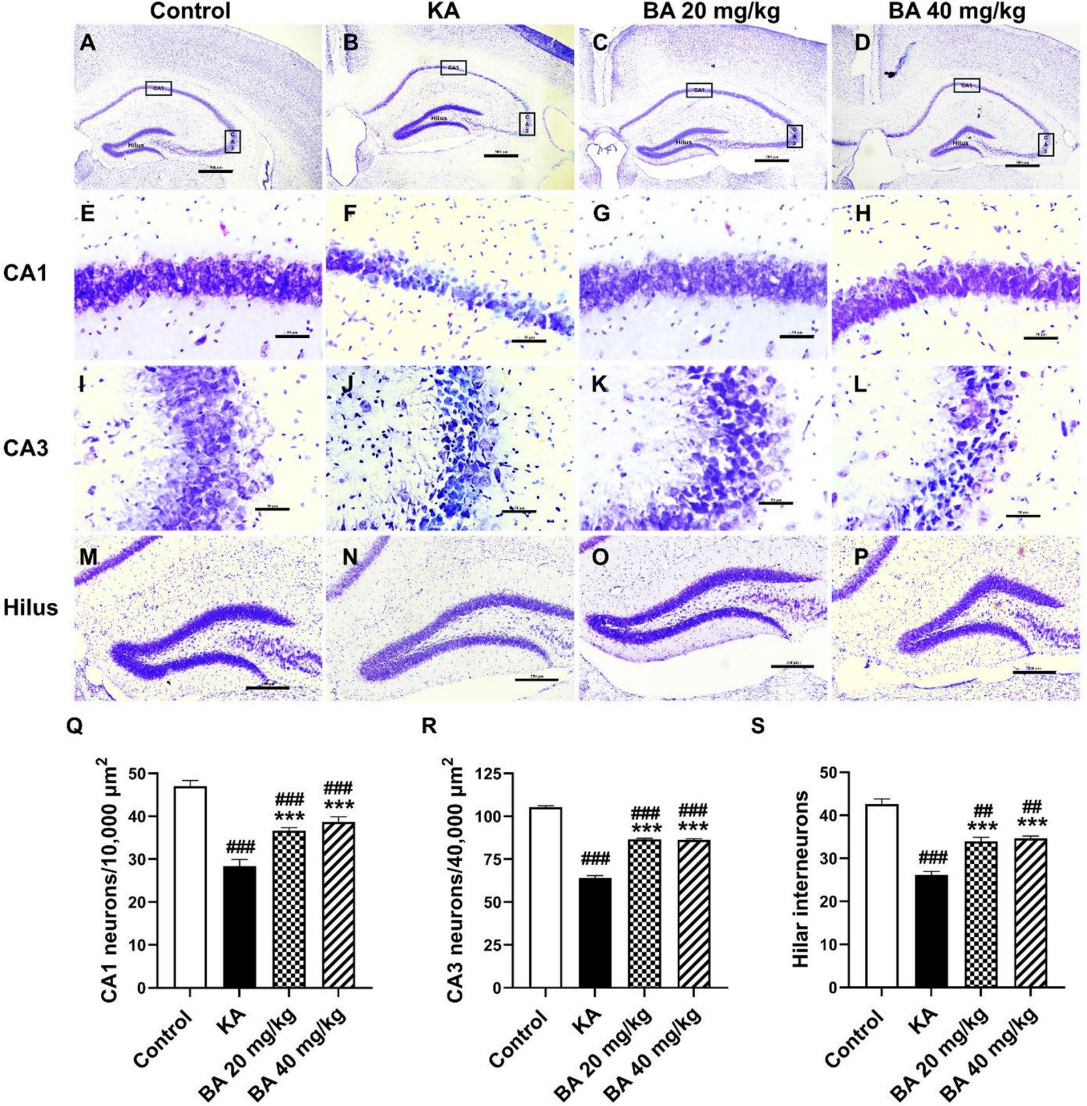
BA exerts neuroprotective effects against KA-induced neuronal cell death within the hippocampus. Nissl stain of the hippocampus highlights the CA1, CA3, and hilar regions used for neuronal quantification (**A**–**D**). CA1 sub-field (**E**–**H**), CA3 sub-field (**I**–**L**), and the hilus of the dentate gyrus (**M**–**P**). Quantifications of CA1 neurons (**Q**), CA3 neurons (**R**), and the hilar interneurons (**S**). Scale bar in A-D = 500 μm, E-L = 50 μm, and M-*P* = 250 μm. Data are expressed as mean ± SEM (*n* = 4 mice per group). ##*p* < 0.01, ###*p* < 0.001 versus control group; ***p < 0.001 versus KA group.

Several studies have demonstrated that KA administration-induced status epilepticus results in the death of neurons in the CA1 and CA3 sub-fields of the hippocampus, and interneurons in the hilus of the dentate gyrus^3,4^. Fourteen days after KA injection, mice showed morphological changes in the hippocampus assessed by Nissl staining (Fig. 3A, P). Mice treated with KA alone revealed a significant reduction in the numbers of CA1 neurons when compared to control mice (*p* < 0.001). However, the number of CA1 neurons were significantly increased following treatment with BA at 20 or 40 mg/kg as compared to mice treated with KA alone (Fig. 3Q). The neuroprotective effect of BA against KA-induced neuronal loss was also confirmed by CA3 neuron counts. Mice treated with KA alone revealed a significant decrease in the number of CA3 neurons compared to the control group (*p* < 0.001). In contrast, the number of CA3 neurons was strikingly increased in mice treated with BA at 20 or 40 mg/kg compared to mice treated with KA alone (Fig. 3R).

Hilar interneurons contain Gamma-amino-butyric acid (GABA) and play a crucial role in maintaining the balance of excitation and inhibition. Research indicated that the loss of hilar GABAergic neurons results in reduced inhibition and generalized epileptic seizures^20^. Thus, we also observed KA treatment-induced loss of hilar interneurons. In contrast, treatment with BA at 20 or 40 mg/kg significantly increased the number of hilar GABAergic interneurons compared to the KA alone group (Fig. 3S). These findings indicate that BA exerts neuroprotective effects, leading to an increase in the number of CA1 and CA3 neurons, and hilar interneurons, which is associated with an improvement in cognition, and a reduction in seizure scores.

### BA increases astrocyte reactivity in response to KA-induced seizure

**Fig. 4:**
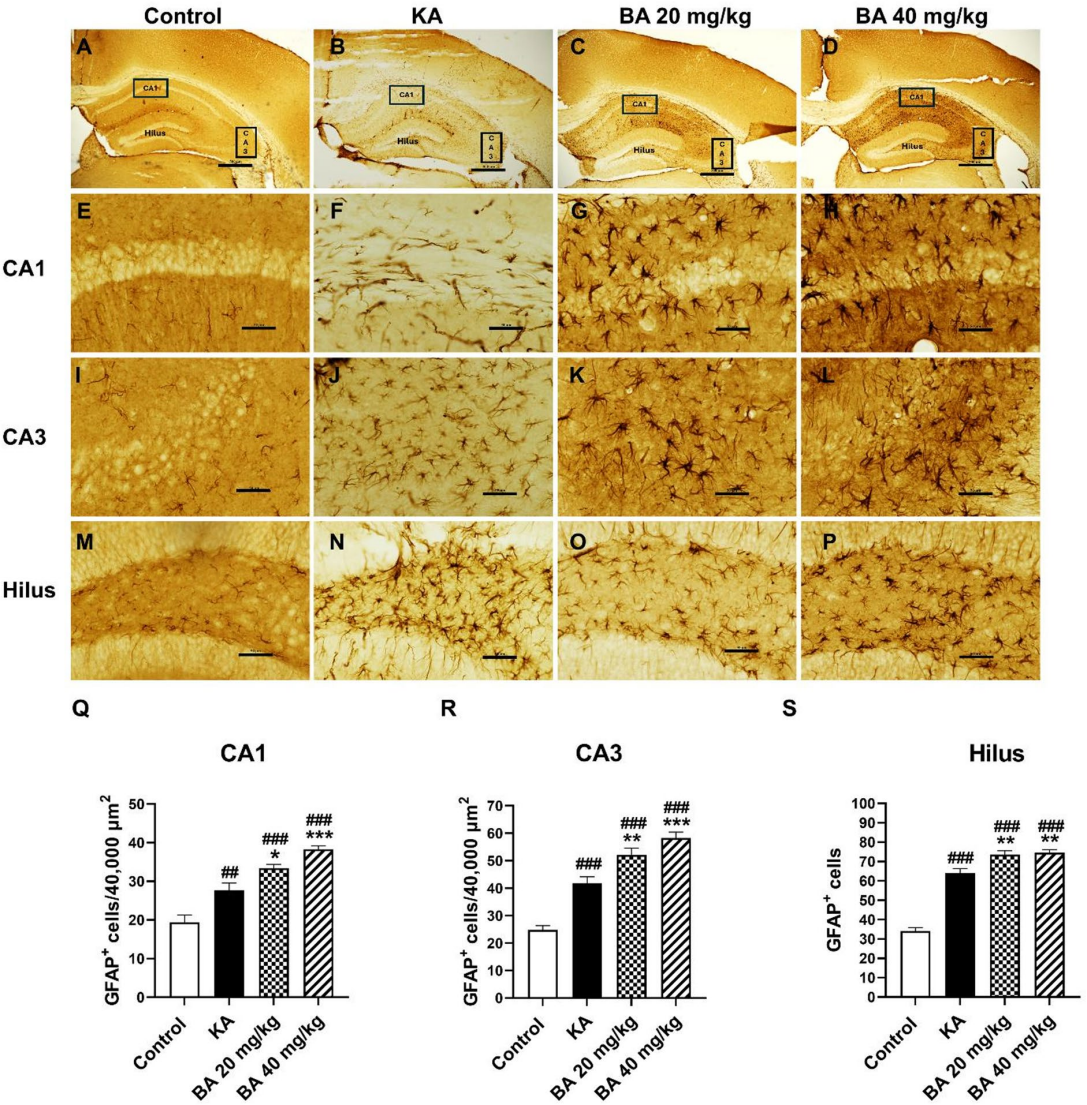
BA enhances astrocytosis within the hippocampus in response to KA-induced neuronal cell death. GFAP stain of the hippocampus marks the CA1, CA3, and hilar regions used for quantification (**A**–**D**). GFAP stains of CA1 sub-field (**E**–**H**), CA3 sub-field (**I**–**L**), and the hilus (**M**–**P**). Quantifications of GFAP-positive cells in the CA1 (**Q**), CA3 (**R**), and the hilus (**S**). Scale bar in A–D = 500 μm, E–L = 50 μm, and M–P = 250 μm. Data are expressed as mean ± SEM (*n* = 4 mice per group). ##*p* < 0.01, ###*p* < 0.001 versus control group; *p < 0.05, **p < 0.01, ***p < 0.001 versus KA group.

It is well established that KA administration leads to morphological alterations and proliferation of astrocytes, known as astrocytosis. Although reactive astrocytes promote scarring in response to brain trauma, evidence shows that astrocytes promote neuronal survival by secreting neurotrophic factors such as nerve growth factor (NGF), brain-derived neurotrophic factor (BDNF), and glial cell line-derived neurotrophic factor (GDNF)^6,21^. Additionally, the activation of astrocytes diminish neocortical seizures via enhancing Na^+^-K^+^-ATPase activity^22^. In this study, we used GFAP staining to label reactive astrocytes. Mice treated with KA revealed morphological changes and a significant increase in the number of GFAP-immunopositive astrocytes compared to the control group (Fig. 4A, P). Notably, BA treatments at 20 or 40 mg/kg showed a significant increase in the number of GFAP-positive astrocytes in the CA1 region as compared to the KA alone group (Fig. 4Q). Similar to the CA1 sub-field, the CA3 sub-field of the BA-treated mice revealed a significant increase in the number of GFAP-positive astrocytes as shown in Fig. 4R. Furthermore, an increase in GFAP-positive astrocytes was also confirmed in the hilus. The quantification demonstrated that the number of GFAP-positive astrocytes significantly increased following BA treatments at 20 or 40 mg/kg compared to KA alone group (Fig. 4S). Taken together, the findings suggest that BA enhances astrocytosis in response to KA-induced neuronal cell death which may contribute to neuroprotection and reduced epileptic seizures.

### BA suppresses microglial activation in response to KA-induced seizure

**Fig. 5:**
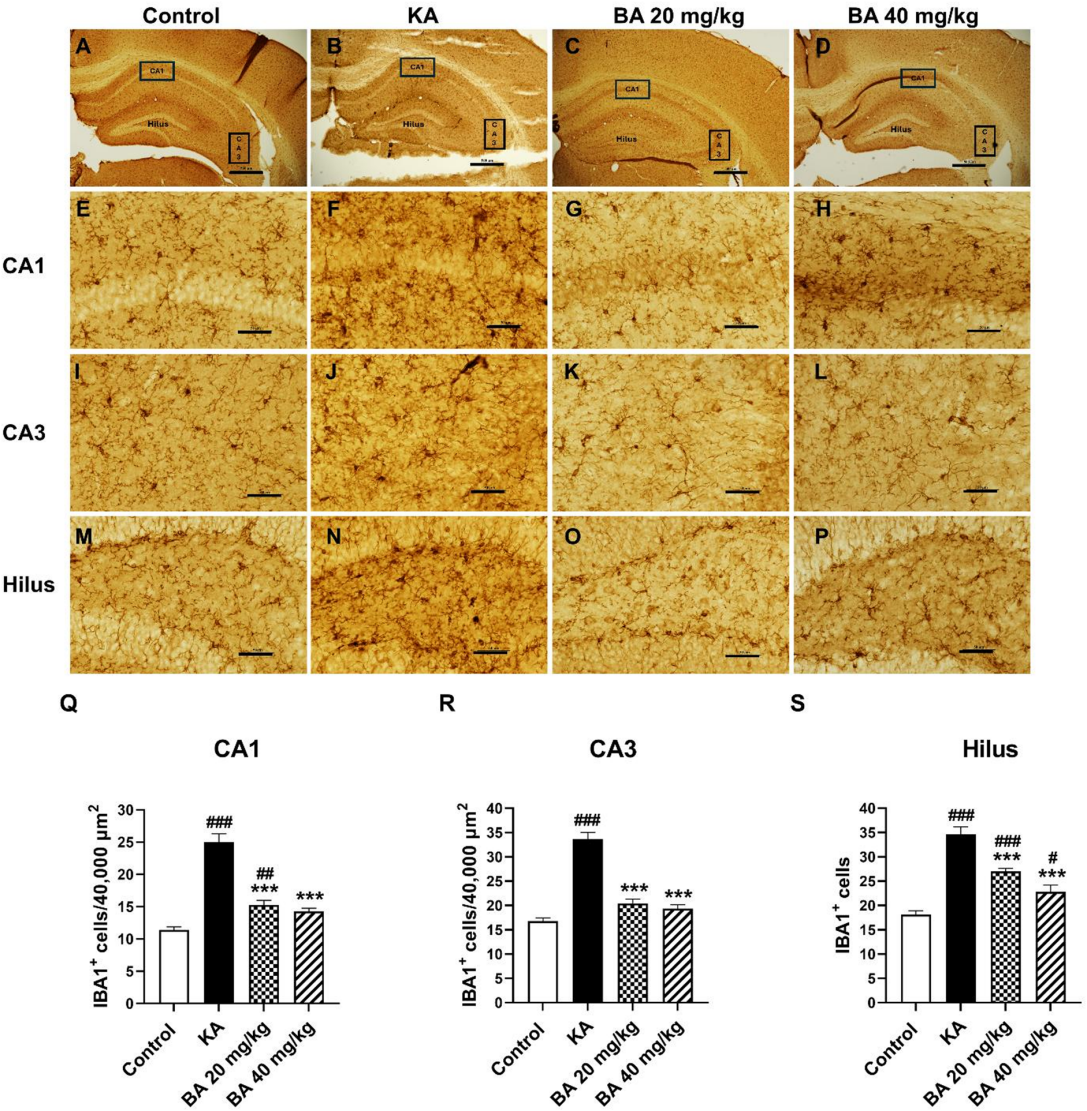
BA suppressed microglial activation within the hippocampus after KA treatment as demonstrated by IBA1 immunoreactivity in the CA1, CA3, and hilus regions of the hippocampus (**A**–**D**). IBA1 stains of CA1 sub-field (**E**–**H**), CA3 sub-field (**I**–**L**), and the hilus (**M**–**P**). Quantifications of IBA1-positive cells in the CA1 (**Q**), CA3 (**R**), and the hilus (**S**). Scale bar in A–D = 500 μm, E––L = 50 μm, and M–P = 250 μm. Results are expressed as mean ± SEM (*n* = 4 mice per group). #*p* < 0.05, ##*p* < 0.01, ###*p* < 0.001 versus control group; ***p < 0.001 versus KA group.

Hyperproliferation and morphological changes are also observed in microglia, which exhibit enlarged cell bodies with thicker and shorter processes^23^. Prolonged expression of reactive microglia in KA-induced epilepsy contributes to the production of proinflammatory cytokines such as tumor necrosis factor alpha (TNF-α), interleukin-1β, and interleukin-6 resulting in neuronal loss and the promotion of epileptic seizures^24–26^. In this study, we investigated whether BA could prevent KA-induced microglial activation at fourteen days after KA injection. After treatment with KA, the number of IBA1-positive microglia was significantly increased in the hippocampus of KA-treated mice compared to mice in the control group (Fig. 5A, P). However, BA treatments at 20 or 40 mg/kg significantly reduce the number of IBA1-positive microglia in the CA1, CA3 and hilar areas compared to the KA lone group. The numbers of IBA1-positive microglia in the CA1 were reduced as well (Fig. 5Q). Similar to the CA1 region, the treatment of BA at 20 or 40 mg/kg markedly reduced the number of IBA1-positive microglia in the CA3 to 20.40 ± 0.91 (*p* < 0.001) and 19.33 ± 0.83 (*p* < 0.001), respectively, compared to mice in KA alone group (Fig. 5R). The prevention of microglial activation by BA was also confirmed in the hilar area; quantification showed a significant reduction in IBA1-positive microglia in the BA treatment groups at 20 or 40 mg/kg compared to the KA alone group (Fig. 5S). These findings demonstrate that KA-induced microglial activation was alleviated by BA treatment, which may have protective effects against KA-induced neuronal cell death and seizure-like behavior.

### BA exerts neuroprotective effects against KA-induced neuronal cell death through the activation of the PI3K/Akt/Nrf2 signaling pathway

**Fig. 6:**
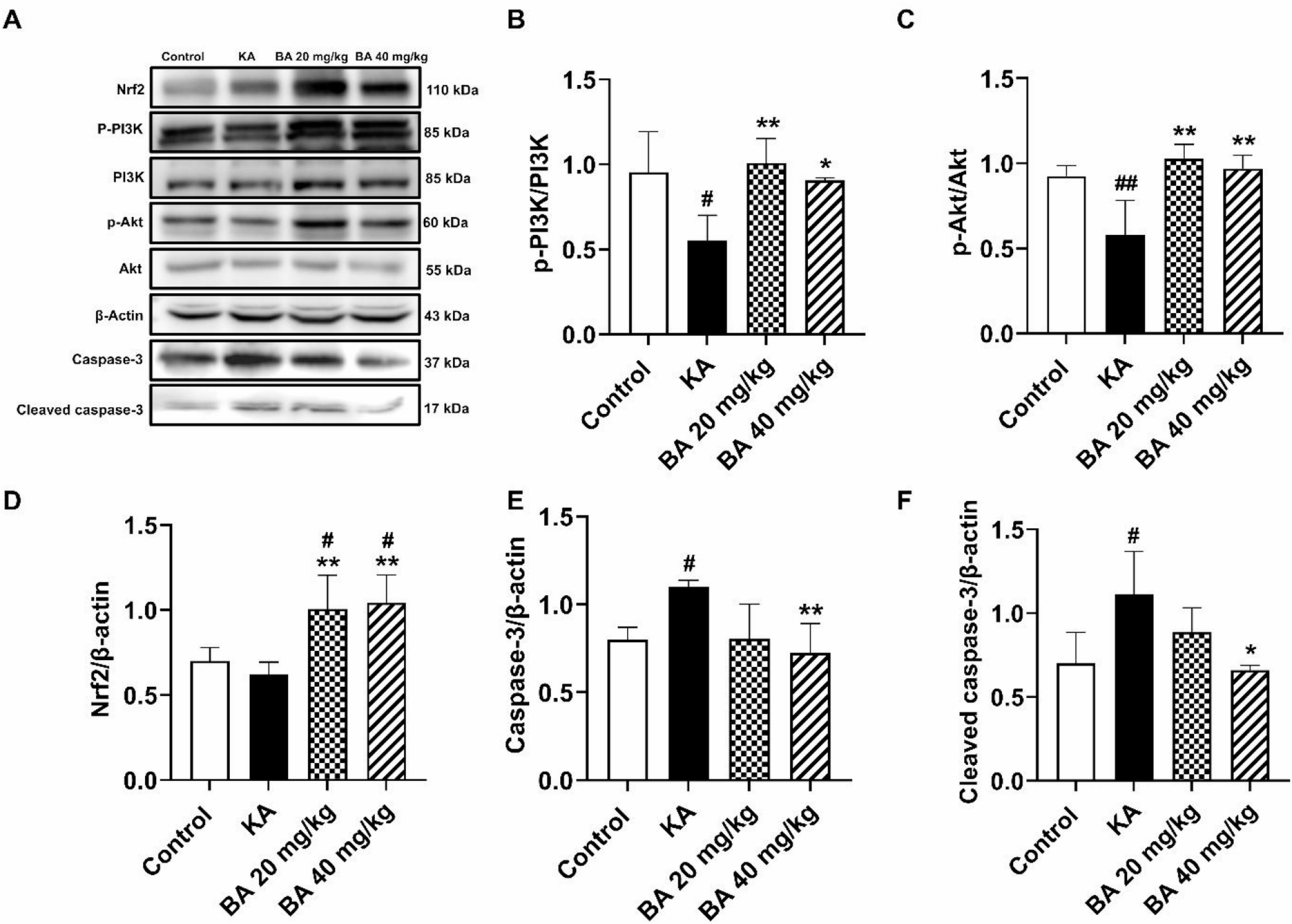
BA treatment activates the PI3K/Akt/Nrf2 signaling pathway while reducing the expression of apoptotic markers after KA treatment. The expression of all proteins was detected by western blotting (**A**). Quantification of the p-PI3K/PI3K ratio (**B**), and the p-Akt/Akt ratio (**C**). The levels of Nrf2 (**D**), caspase-3 (**E**), and cleaved caspase-3 (**F**) were normalized to β-actin. Data are expressed as mean ± SD (*n* = 4 mice per group). #*p* < 0.05, ##*p* < 0.01 versus control group; *p < 0.05, **p < 0.01 versus KA group.

The phosphatidylinositol 3-kinase/protein kinase B (PI3K/Akt) pathway plays a crucial role in the promotion of cell growth, survival, and proliferation. The activation of this pathway leads to a signaling-induced activation of Nrf2, which in turn regulates antioxidant enzymes, including SOD, CAT, and GSH resulting in the prevention of neuronal apoptosis and seizure-like behavior^27–29^. Therefore, we verified whether BA treatment could activate the PI3K/Akt/Nrf2 signaling pathway in response to KA-induced seizures. Western blotting showed that mice treated with BA at 20 or 40 mg/kg demonstrated significantly increased ratios of p-PI3K/PI3K in the hippocampus as compared with the KA alone group (Fig. 6A, B). In addition, the treatment of BA at 20 or 40 mg/kg subsequently resulted in a significant increase in the expression of p-Akt/Akt in the hippocampal tissue compared to the mice treated with KA alone (Fig. 6A, C). We further investigated the expression levels of Nrf2 in the hippocampal tissue after KA-induced epilepsy. Our data demonstrate that treatment with BA at 20 or 40 mg/kg significantly increased the expression of Nrf2 compared with the KA alone group (Fig. 6A, D). These results indicate that BA diminished KA-induced oxidative stress and neuronal cell death by activating the PI3K/Akt/Nrf2 signaling pathway.

### BA attenuates KA-induced expression of apoptotic markers

Treatment with KA has been shown to elevate the expression of caspase-3, an apoptosis-activated enzyme leading to neuronal cell death^30,31^. Our results demonstrate that treatment with KA for 14 days significantly increased the levels of caspase-3 and cleaved (activated) caspase-3 in the hippocampal tissue compared to the control group (*p* < 0.05). However, the level of activated caspase-3 was significantly lower in the hippocampal tissue of mice treated with BA at 40 mg/kg than in the KA alone group (Fig. 6A, E). In addition, the level of cleaved caspase-3 was significantly reduced in mice treated with BA at 40 mg/kg compared to the KA alone group (Fig. 6A, F). These findings support the hypothesis that BA possesses anti-apoptotic effects against KA-induced neuronal cell death.

## Discussion

In this study, our findings show that BA exerts neuroprotective effects in a mouse model of KA-induced epilepsy by inhibiting oxidative stress, neuronal death and microglia activation in the hippocampus. The systemic administration of KA triggers seizure activity, which leads to a large production of ROS, causing ROS-induced DNA damage, protein oxidation, and lipid peroxidation, and subsequent seizure-induced hippocampal cell death^32,33^. In the current study, BA treatment demonstrates increased antioxidant enzyme levels including SOD, GSH, and CAT, and decreased the level of lipid peroxidation marker in the hippocampal tissue. Interestingly, the appearance of increased antioxidant enzyme activity following BA treatment is associated with a reduction in seizure scores and an enhancement of cognitive function. KA-induced excitotoxicity results in neuronal cell loss in the CA1, CA3 areas, and in the dentate hilus, leading to memory deficit and seizure severity^14,34^. In this study, cresyl violet staining revealed the neuroprotective effects of BA in the CA1, CA3 areas, and in the hilus against KA-induced cell damage. Taken together, these finding results suggest that BA plays a critical role in mitigating KA-induced oxidative stress and neurodegeneration, which may serve as a potent and novel therapeutic agent in the treatment of epilepsy.

It is well established that administration of KA results in astrocyte and microglia activation, which is known as gliosis. The activation of astrocytes following KA injection has been shown to protect hippocampal neurons from death and increase GABA levels in the hippocampus^35^. Furthermore, astrocytes can prevent neuronal excitotoxicity by uptake of extracellular glutamate via excitatory amino acid transporters (EAAT)^1^.

In the antioxidant defense system, astrocytes protect neurons from oxidative stress by producing GSH through the activation of nuclear factor erythroid 2-related factor 2 (Nrf2)^36^. In this animal model of KA-induced epilepsy, the number of astrocytes is enhanced by BA following KA-induced epilepsy, which may lead to the observed increase in seizure threshold and a decrease of neuronal death in the hippocampus and suggest that the neuroprotective effect of BA is at least partially attributable to reactive astrocytosis. Microglia is also activated in response to KA -induced excitotoxicity and has been shown to release ROS and pro-inflammatory cytokines (TNF-α, IL-1, and IL-6), which result in progressive neuronal degeneration^37,38^. Conversely, inhibiting microglial activation significantly reduces oxidative stress and pro-inflammatory cytokines in the hippocampus, which are a cause of neuronal damage^39^. Recently, numerous studies demonstrated anti-inflammatory and antioxidant activities of BA in lipopolysaccharide-stimulated microglial activation, thereby leading to decreased levels of IL-1β, IL-6, TNF-α, and ROS^9,13,40^. In this study, we demonstrated that BA treatment significantly decreased the microglial activation, suggesting that BA may have a therapeutic effect against KA-induced excitotoxicity by inhibiting microglial activation.

Furthermore, we investigated the impact of BA on expression of proteins in the hippocampus following KA injection using western blotting. The PI3K/Akt signaling pathway plays a crucial role in the neuronal survival and antioxidant defense system. Activation of the PI3K/Akt signaling pathway diminishes the seizure severity and protects against neuronal apoptosis in animal models of epilepsy^29,41^. In addition, PI3K/Akt is upstream of Nrf2, a transcription factor for generating antioxidative enzymes. It has been shown that the upregulation of Nrf2 can significantly increase the levels of antioxidative enzymes and protect hippocampal neurons from injury^28,29^. A previous study has demonstrated that BA reduces oxidative stress via activated Nrf2 signaling, thereby alleviating spinal cord injury at an early stage^42^. Moreover, BA can protect against rotenone-induced Parkinson’s disease via the activation of PI3K/Akt signaling, leading to neuroprotection of dopaminergic neurons^43^. Furthermore, our data reveal the influence of BA on caspase-3, a biomarker for neuronal apoptosis. It is known that caspase-3 is activated by KA administration, leading to prolonged seizure activity and neuronal apoptosis^44,45^. In contrast, inhibition of caspase-3 attenuates KA-induced apoptotic cell death following status epilepticus^44^. In a rat model of ischemic stroke, treatment of BA can reduce neuronal damage by alleviating caspase-3 levels^46^. In the model of doxorubicin- induced cognitive deficits and neuroinflammation, BA reduced DOX-induced apoptosis in the hippocampus, demonstrated by a 26% decrease in hippocampal caspase-3 content^47^. Moreover, in primary hippocampal neuron cultures, BA mitigated H_2_O_2_-induced elevations in Caspase-3 activity, thereby promoting the survival of the hippocampal neurons^48^. In the current study, KA administration exhibited reduced activation of PI3K/Akt/Nrf2, and increased levels of caspase-3 (total and activated). Notably, BA treatment was able to restore the PI3K/Akt/Nrf2 signaling pathway and suppress the expression of caspase-3. Our findings highlight that BA may attenuate seizure severity and hippocampal neuronal damage through activation of the PI3K/Akt/Nrf2 signaling pathway and inhibition of caspase-3 expression.

**Fig. 7:**
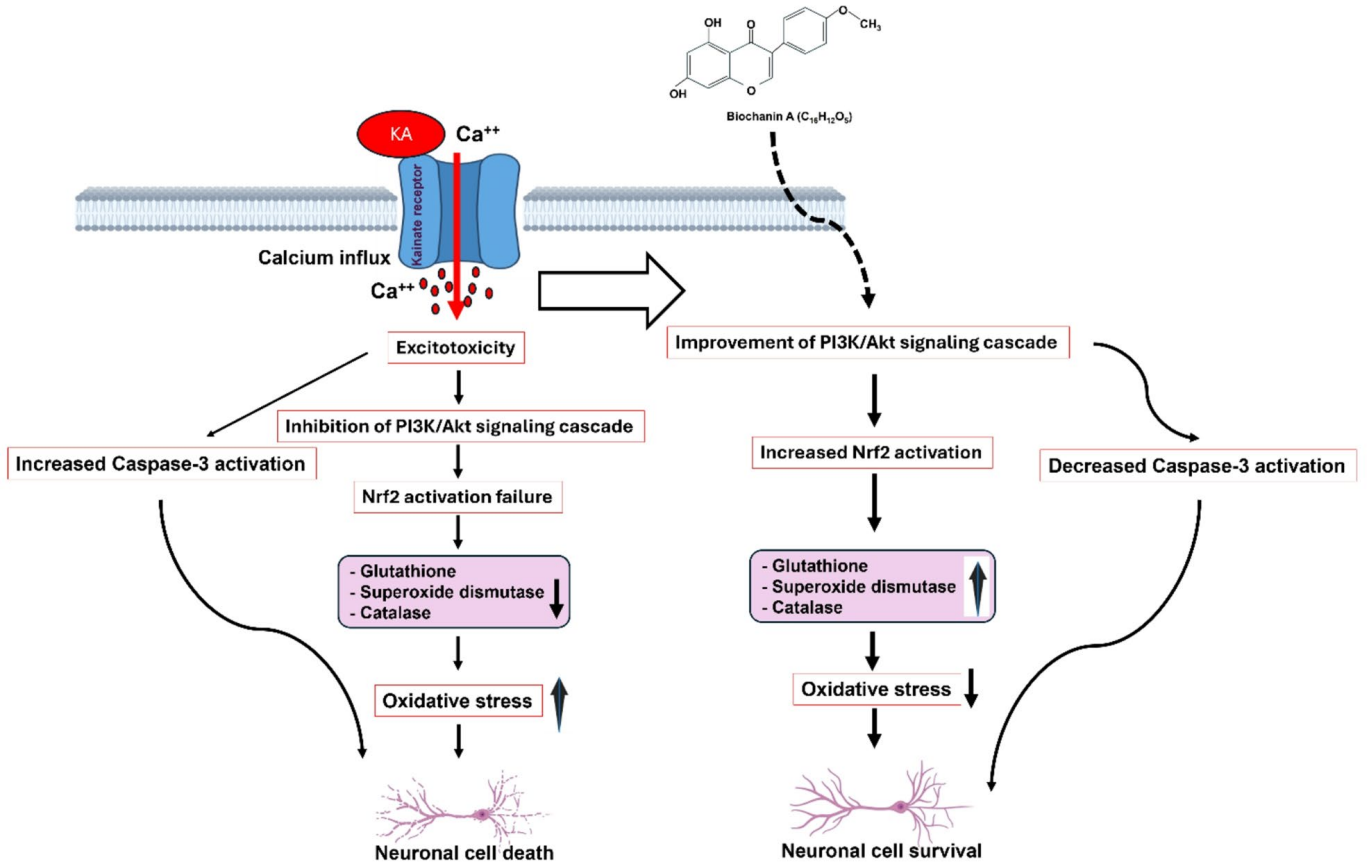
Presumptive mechanism of BA in KA-induced excitotoxicity. Activation of kainate receptors by kainic acid evokes excessive Ca^2+^ entry into the neuron, leading to excitotoxicity. This process inhibits the PI3K/Akt signaling cascade, which in turn leads to Nrf2 activation failure. The consequence is the production of caspase-3 activation and oxidative stress, ultimately resulting in neuronal cell death. BA exerts a neuroprotective effect most likely via an upregulation of the PI3K/Akt/Nrf2 signaling pathway, leading to improved antioxidant enzyme levels and neuronal survival.

## Conclusion

In conclusion, we demonstrate that BA treatment attenuates seizure scores and hippocampal neuron loss in a mouse model of KA-induced epilepsy. Our protein expression data suggest that BA activates the PI3K/Akt/Nrf2 signaling pathway, leading to an increase in antioxidant enzyme levels, thereby improving neuronal survival. The anti-apoptotic effect of BA is presumably mediated by the inhibition of caspase-3 expression (Fig. 7). These findings indicate that BA could be a therapeutic agent for epilepsy or for other neurodegenerative diseases where oxidative stress plays a crucial role.

## Supplementary Information

Below is the link to the electronic supplementary material.

Supplementary Material 1

## Data Availability

The datasets used and/or analyzed during the current study are available from the corresponding author on reasonable request.
