# Supplementary material for: Biochanin A prevents neurodegeneration and oxidative stress in a kainic acid model of epilepsy by activating the PI3K/Akt/Nrf2 signaling pathway
Source: Sci Rep. 2025 Nov 13;15:39842. doi: 10.1038/s41598-025-23414-z (PMC12615672; doi:10.1038/s41598-025-23414-z)

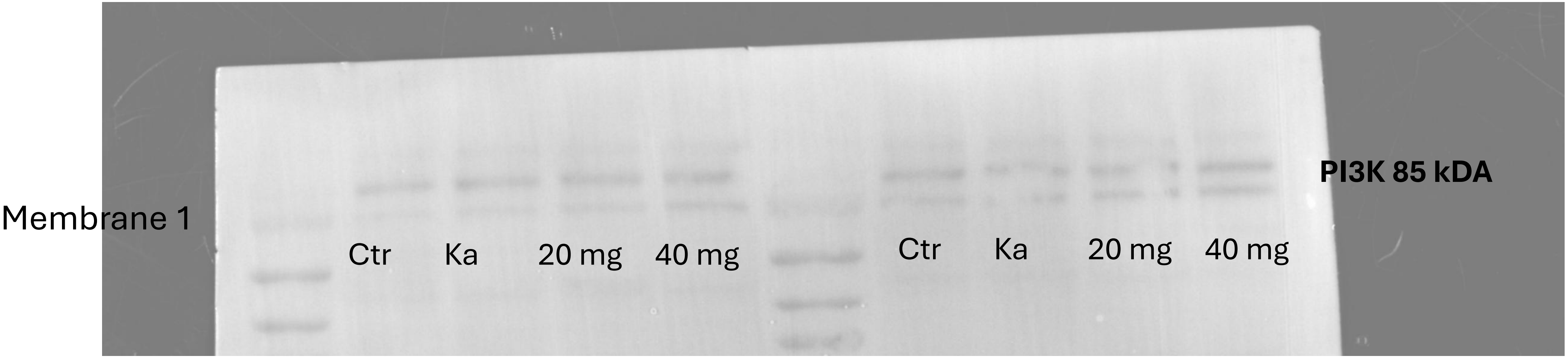
Membrane 1
Ctr
Ka
20 mg
40 mg
Ctr
Ka
20 mg
40 mg
PI3K 85 kDA

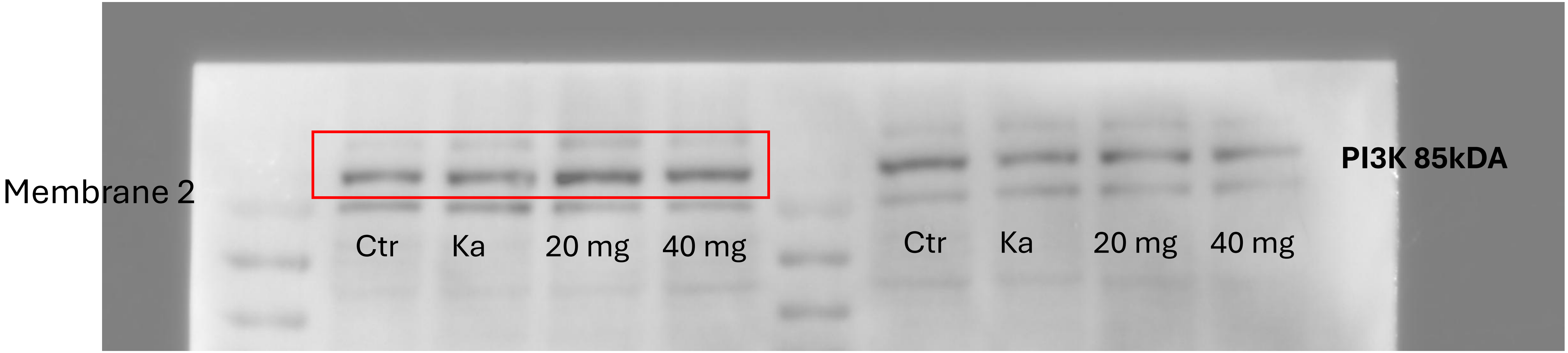
Membrane 2
Ctr
Ka
20 mg
40 mg
Ctr
Ka
20 mg
40 mg
PI3K 85kDA

membrane1

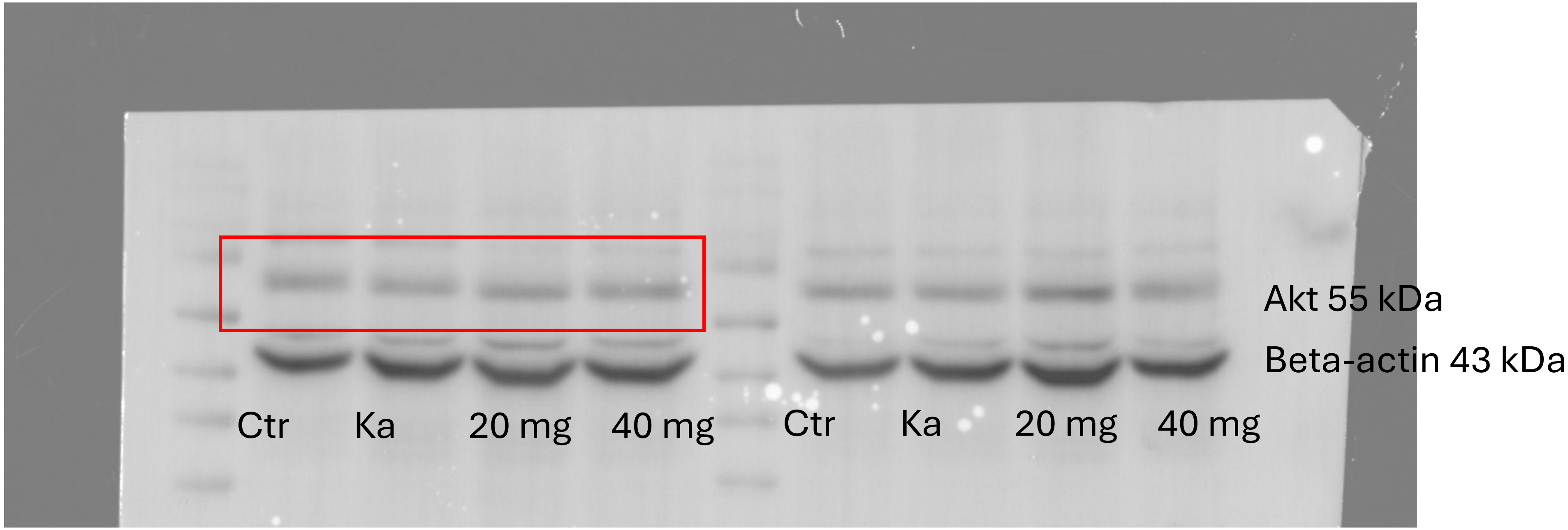

membrane2

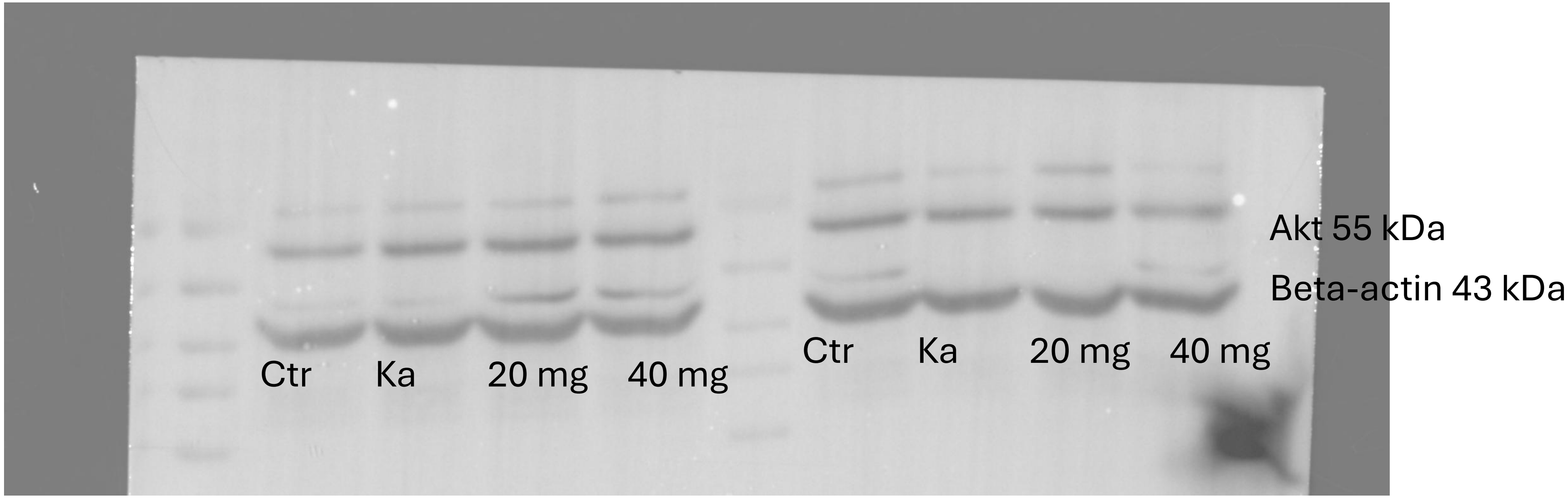

membrane1

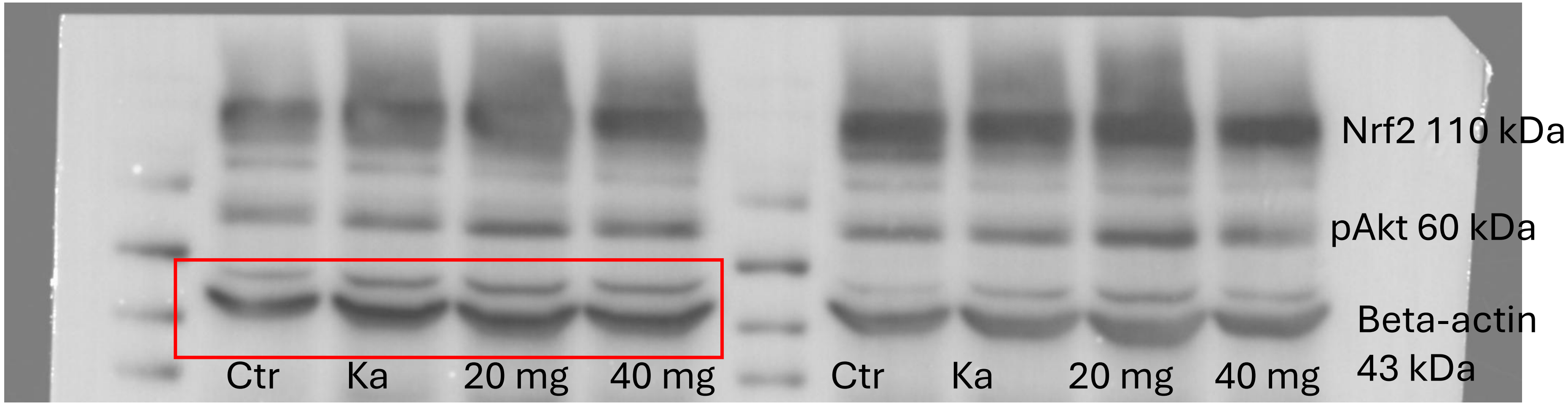

membrane2

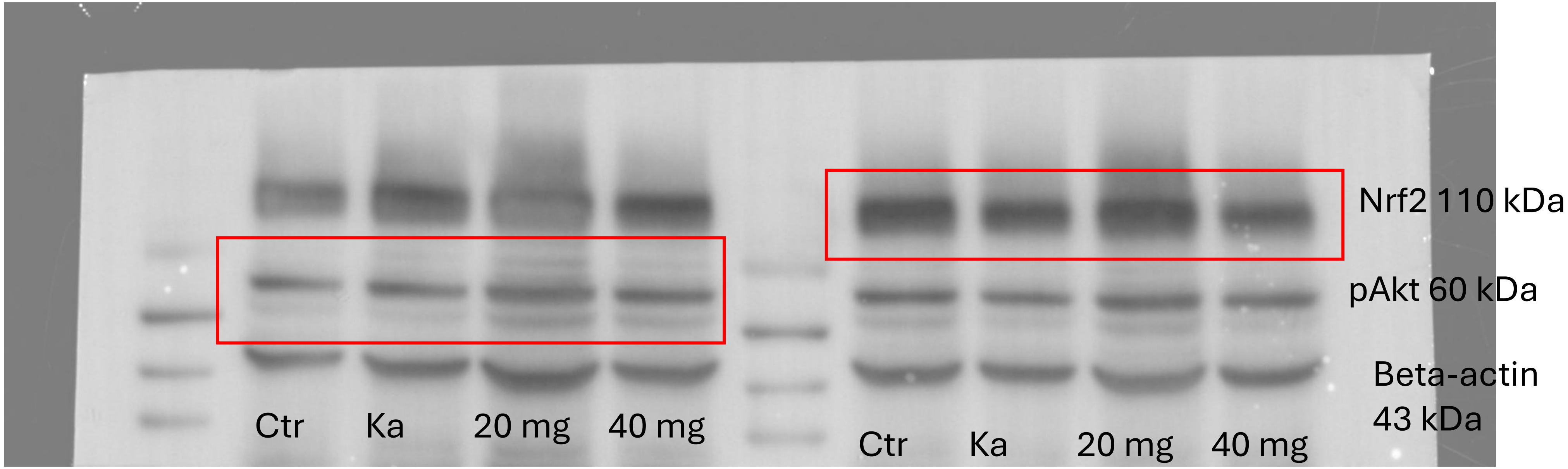

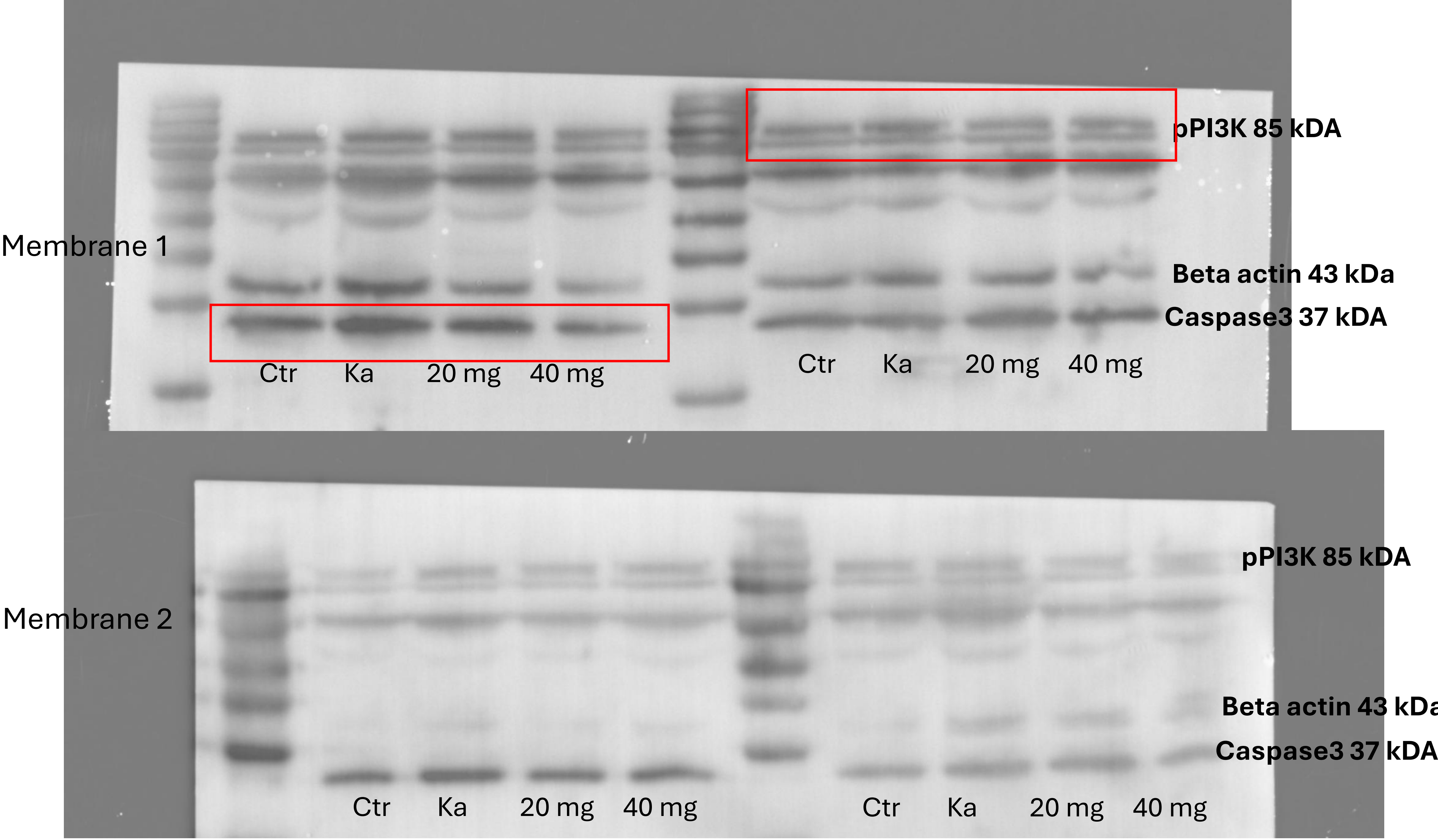
Membrane 1
pPI3K 85 kDA
Beta actin 43 kDa
Caspase3 37 kDA
Ctr
Ka
20 mg
40 mg
Ctr
Ka
20 mg
40 mg
Membrane 2
pPI3K 85 kDA
Beta actin 43 kDa
Caspase3 37 kDA
Ctr
Ka
20 mg
40 mg
Ctr
Ka
20 mg
40 mg

Membrane 1

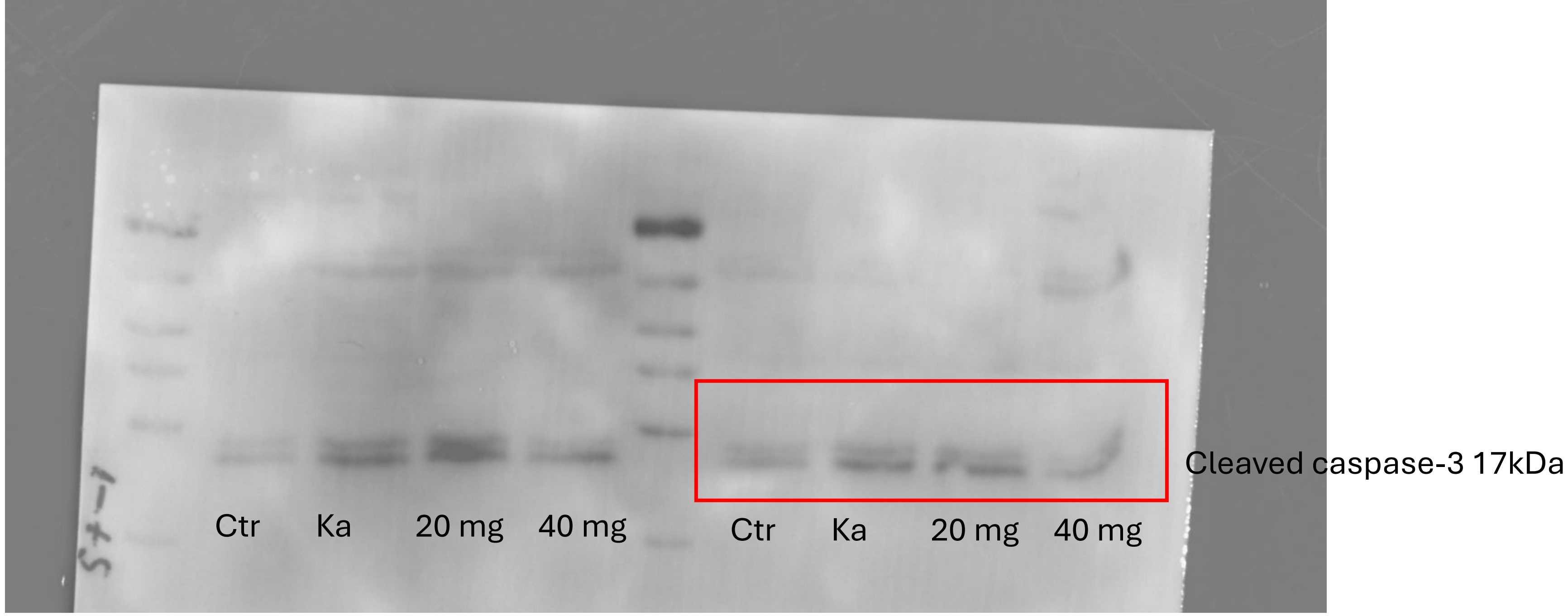

Membrane 2

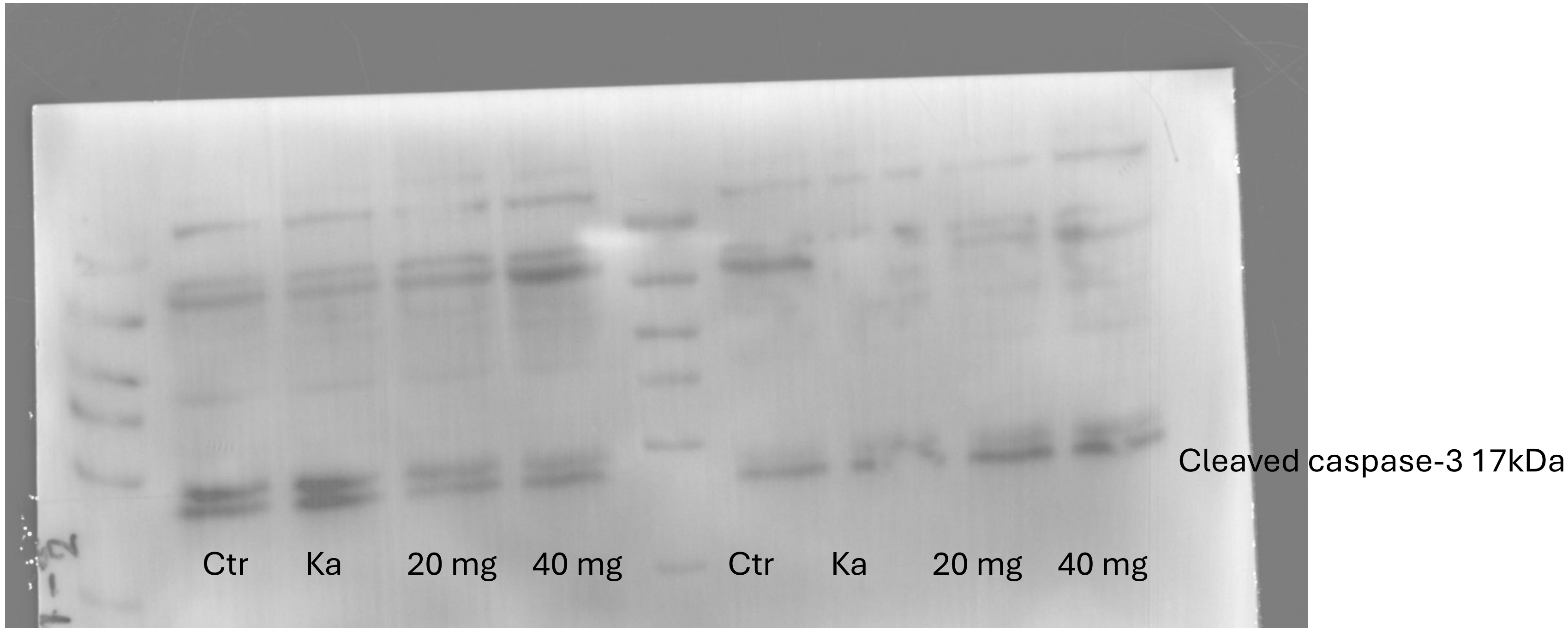

Supplement: Supplementary file 1 — Supplementary Material 1 [file 41598_2025_23414_MOESM1_ESM.pdf]
